# Supplementary material for: Identification of Structural Variation in Chimpanzees Using Optical Mapping and Nanopore Sequencing
Source: Genes (Basel). 2020 Mar 4;11(3):276. doi: 10.3390/genes11030276 (PMC7140787; doi:10.3390/genes11030276)
Supplement: Supplementary file 1 [file genes-11-00276-s001.zip › SupplementaryFiles/SupFigures.pdf]

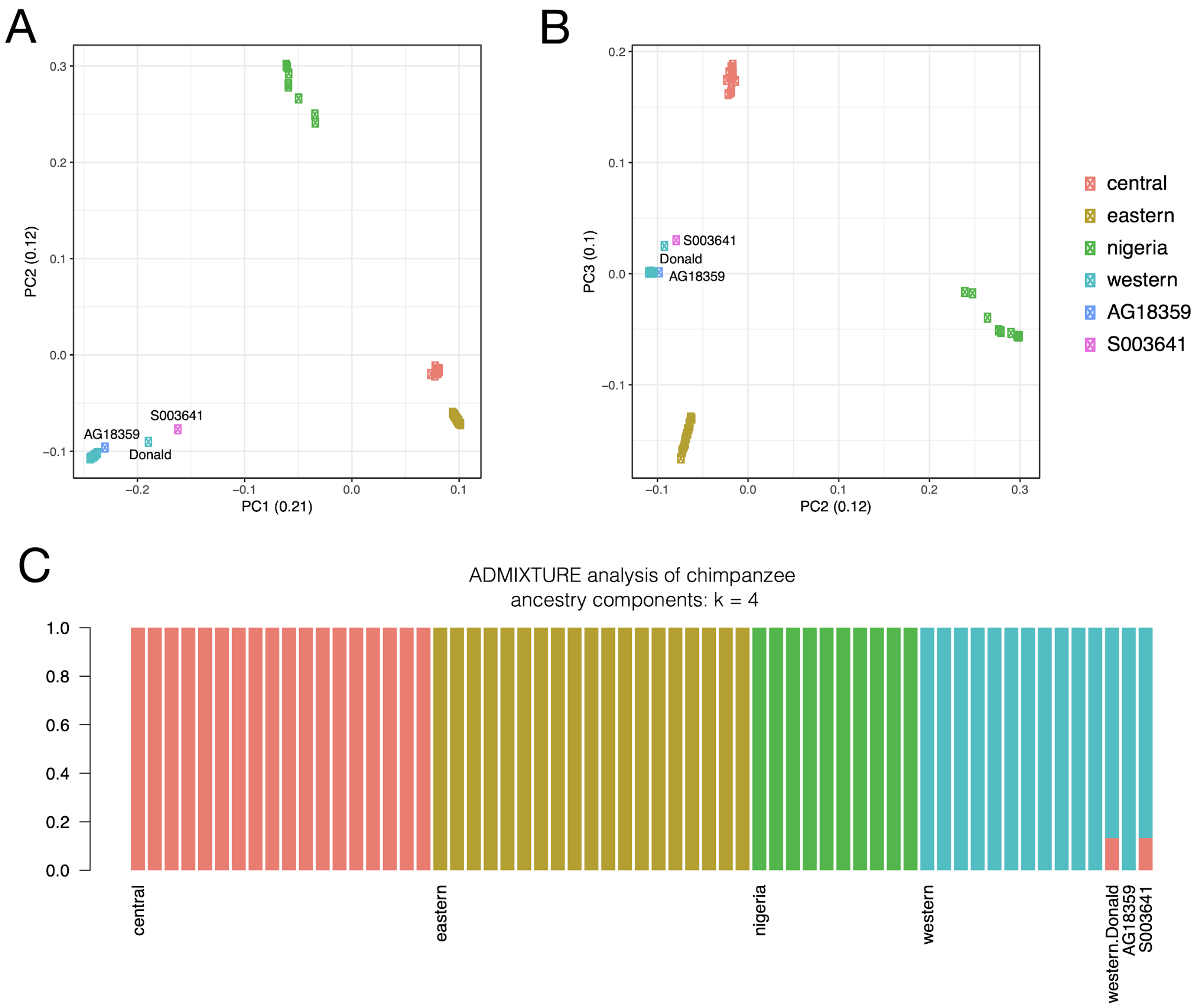

Figure S1. Chimpanzee subspecies identification. (A) and **(B)** PC analysis of chimpanzee genetic diversity. Both of the newly sequenced cell lines were projected onto PCs inferred from the 59 chimpanzees presented in de Manuel et al. (2016) [32]. Both cell lines show closest affinity to western chimpanzees (*Pan troglodytes verus*). While AG18359 clusters tightly with the western subspecies, S003461 also shows affinity to the central/eastern clade, with PC3 indicating that, like Donald, this cell line was derived from a hybrid individual with central ancestry. Values in parentheses are the proportion of variance explained by each PC. **(C)** ADMIXTURE analyses, assuming four ancestral components (K = 4) confirms the hybrid origin of S003641.

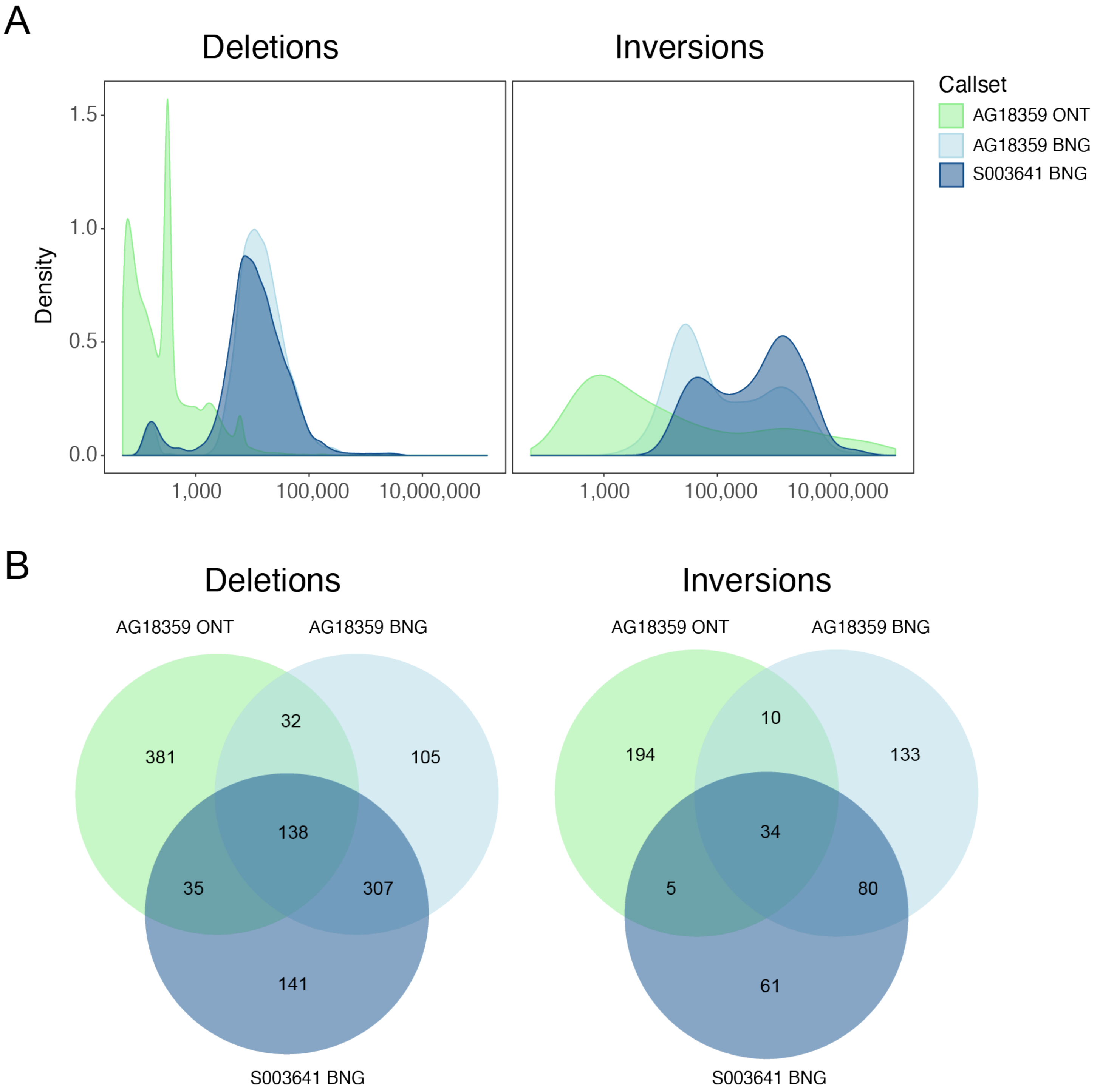

Figure S2. Description of SV discovery set. (**A**) Length distribution (x-axis in bp) of raw SV calls discovered by ONT (green) and BNG (light blue) from AG18359, and BNG from S003641 (dark blue). (**B**) Venn diagram comparing large (≥10 kbp) deletions (left) and inversions (right) discovered for each individual and technology (not to scale). Two variants were considered the same if they have a 50% reciprocal overlap.

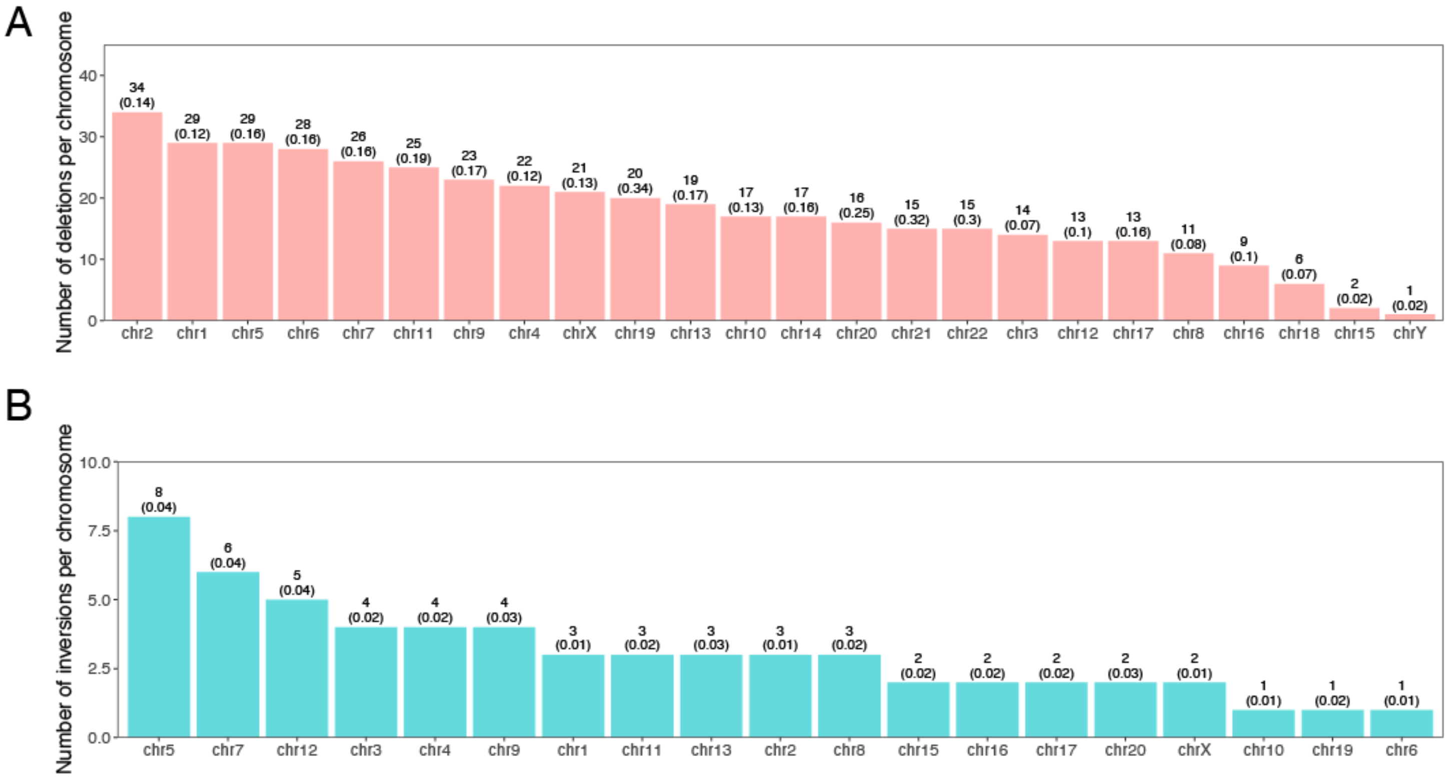

Figure S3. Histogram of identified SV events per chromosome. The number of high-confidence SV events discovered is depicted for (**A**) deletions and (**B**) inversions. The normalized number of events per Mbp for each chromosome is displayed in parentheses.

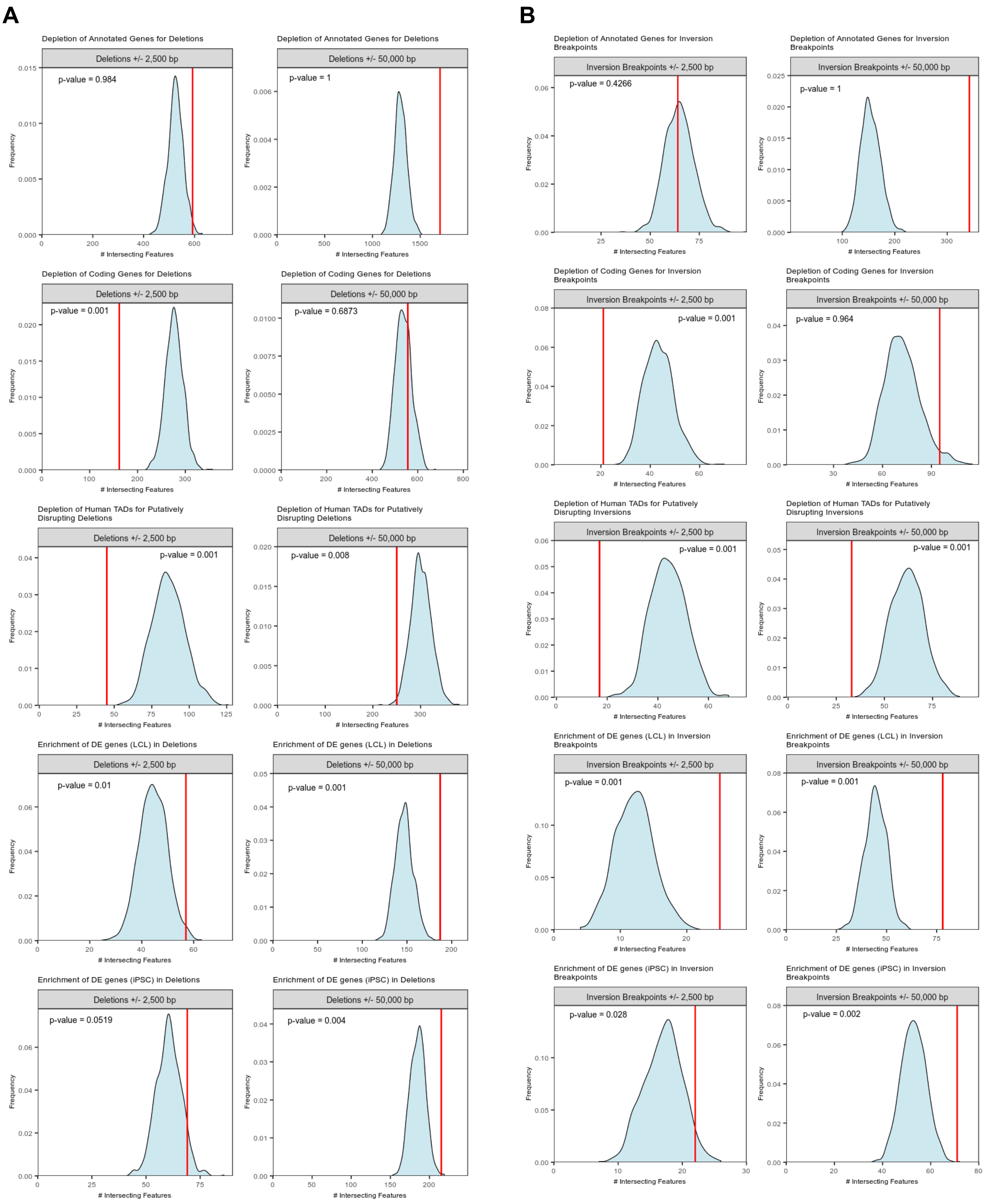

Figure S4. Enrichment/depletion of SV breakpoints for genomic features of interest as determined by permutation testing. Each plot compares the observed count of intersecting features (red vertical line) to a distribution of counts generated from 1000 permuted sets of coordinates (for testing depletion of SVs) or 1000 randomly selected genes from the background list of each DE analysis (for testing enrichment of DE genes in SVs) for (**A**) deletions and (**B**) inversions.

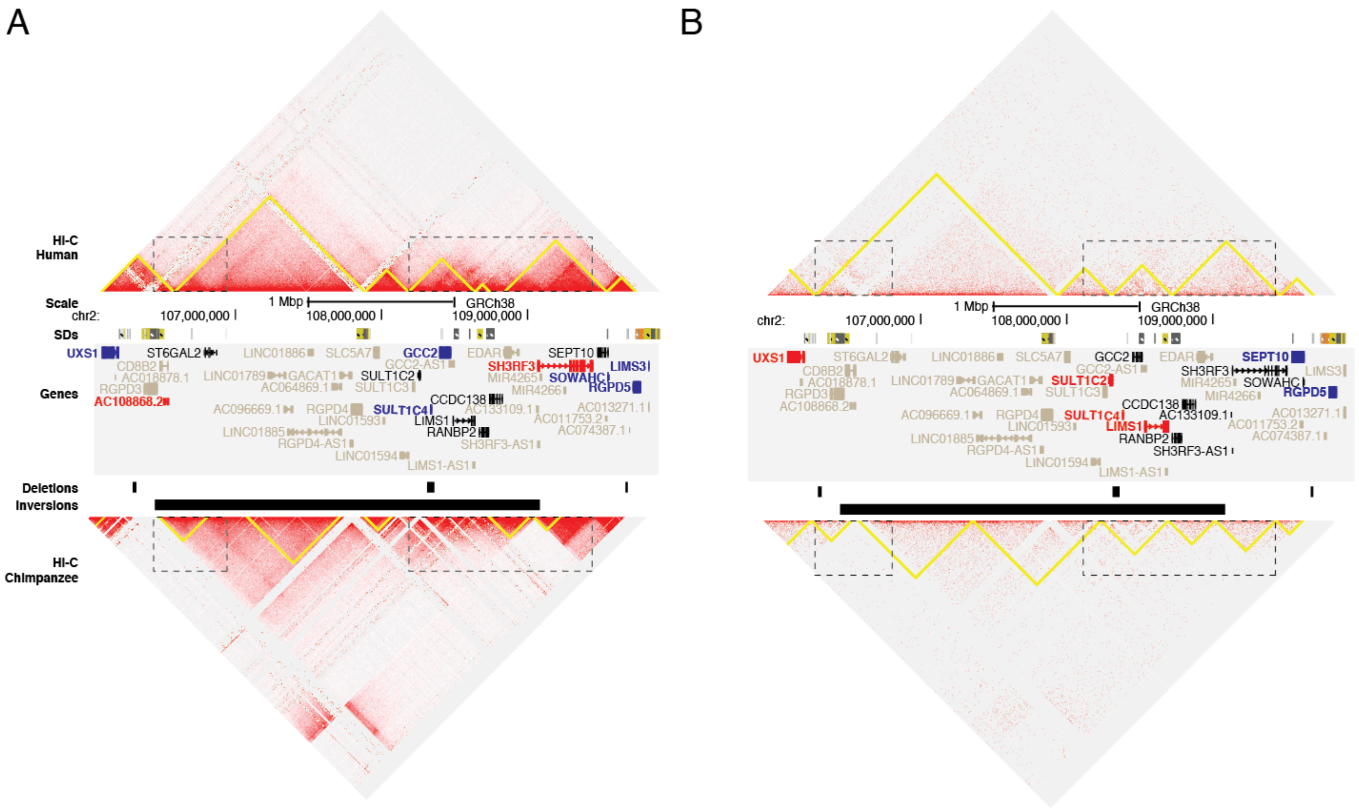

Figure S5. Genome organization of human chromosome 2q12.2-q13. The Hi-C genomic landscape of human (top) and chimpanzee (bottom) are depicted for iPSCs (**A**) and LCLs (**B**) using Juicebox at chr2:106,095,001-109,905,000 (GRCh38). Predicted TADs (yellow triangles) were compared between species, noting differences at SVs (dotted rectangles) including deletions and inversions. SDs are depicted as colored bars, taken from the UCSC Genome Browser track. Genes showing significant DE in chimpanzee versus humans are colored as blue (down in chimpanzee) or red (up in chimpanzee). Genes not included in the DE analysis are gray.

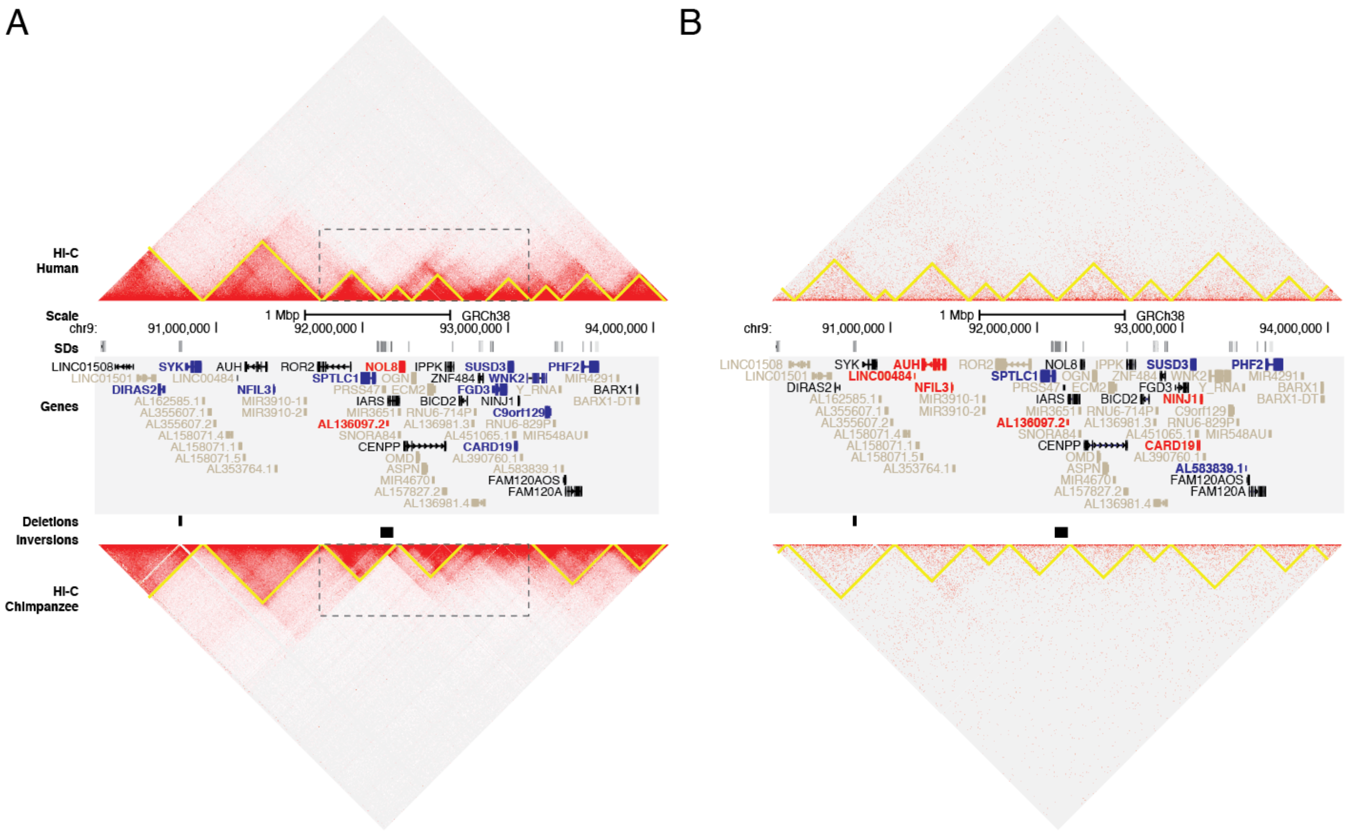

Figure S6. Genome organization of human chromosome 9q22.2-q22.32. The Hi-C genomic landscape of human (top) and chimpanzee (bottom) are depicted for iPSCs (**A**) and LCLs (**B**) using Juicebox at chr9:90,200,001-94,010,000 (GRCh38). Predicted TADs (yellow triangles) were compared between species, noting differences at SVs (dotted rectangles) including deletions and inversions. SDs are depicted as colored bars, taken from the UCSC Genome Browser track. Genes showing significant DE in chimpanzee versus humans are colored as blue (down in chimpanzee) or red (up in chimpanzee). Genes not included in the DE analysis are gray.

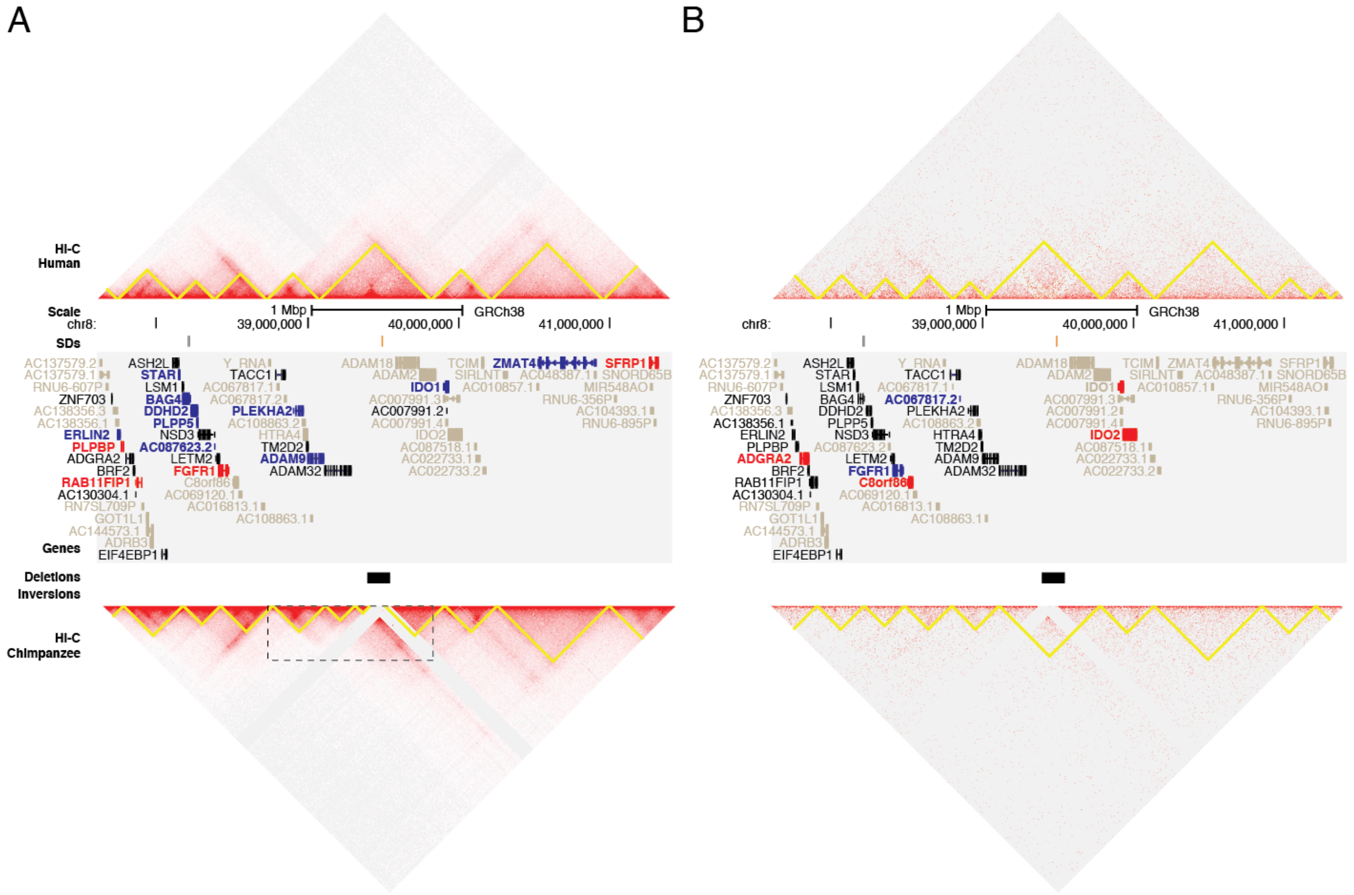

Figure S7. Genome organization of human chromosome 8p11.23-p11.21. The Hi-C genomic landscape of human (top) and chimpanzee (bottom) are depicted for iPSCs (**A**) and LCLs (**B**) using Juicebox at chr8:37,620,001-41,430,000 (GRCh38). Predicted TADs (yellow triangles) were compared between species, noting differences at SVs (dotted rectangle) including deletions and inversions. SDs are depicted as colored bars, taken from the UCSC Genome Browser track. Genes showing significant DE in chimpanzee versus humans are colored as blue (down in chimpanzee) or red (up in chimpanzee). Genes not included in the DE analysis are gray.

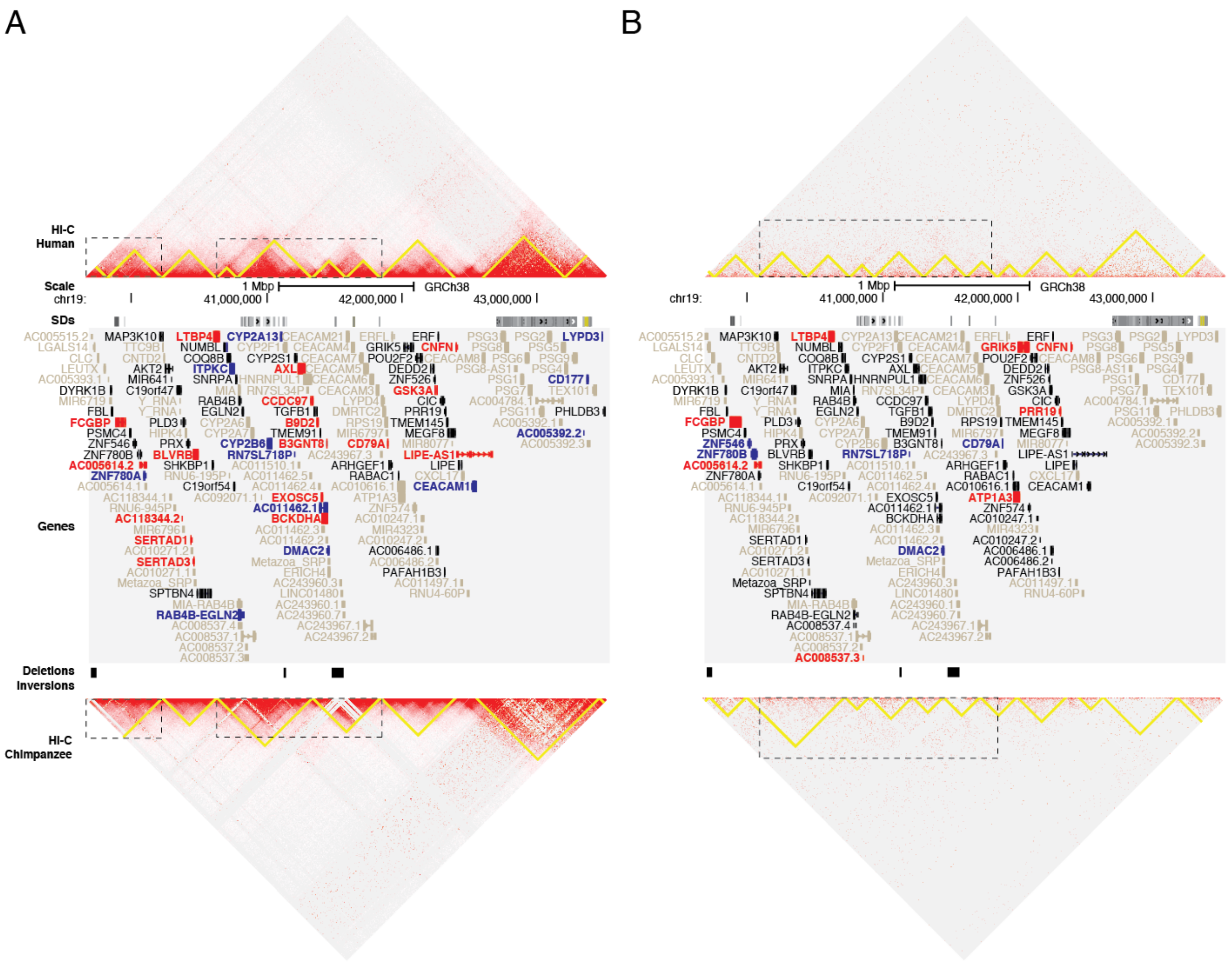

Figure S8. Genome organization of human chromosome 19q13.2-q13.31. The Hi-C genomic landscape of human (top) and chimpanzee (bottom) are depicted for iPSCs (**A**) and LCLs (**B**) using Juicebox at chr19:39,685,001-43,495,000 (GRCh38). Predicted TADs (yellow triangles) were compared between species, noting differences at SVs (dotted rectangles) including deletions (no inversions were identified as this locus). SDs are depicted as colored bars, taken from the UCSC Genome Browser track. Genes showing significant DE in chimpanzee versus humans are colored as blue (down in chimpanzee) or red (up in chimpanzee). Genes not included in the DE analysis are gray.

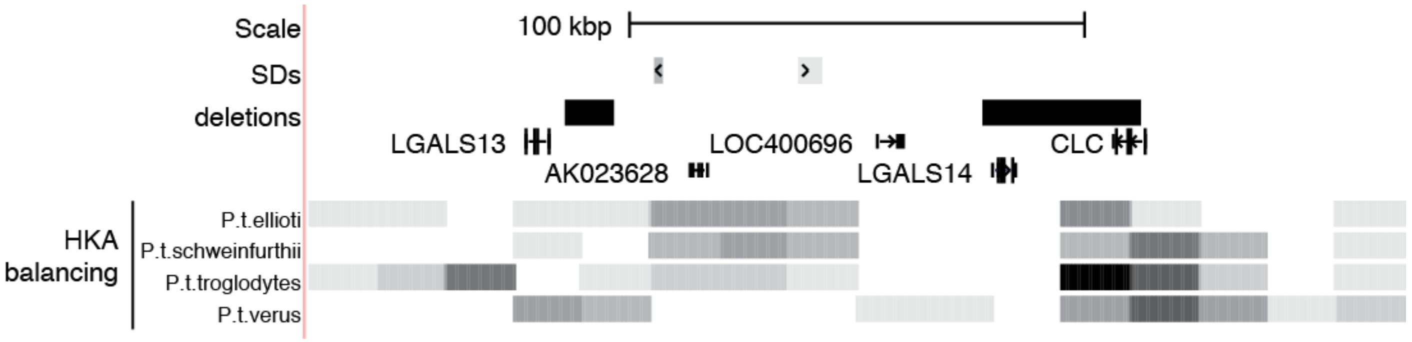

Figure S9. Chimpanzee-specific deletions of the galectin family of genes. Pictured is a UCSC Genome Browser snapshot of human chromosome 19p13.2. The locations of SDs (colored bars), deletions (black bars), and genes are indicated. For each subspecies of chimpanzee (*Pan troglodytes* (*P.t.*)), the -log-*p*-value for the HKA test of balancing selection is depicted as shades of gray in 15-kbp windows (darker shade indicates greater significance) as determined by Cagan et al., 2016 [45] (human reference hg18).
